# Trans-splicing repair of mutant p53 suppresses the growth of hepatocellular carcinoma cells *in vitro* and *in vivo*

**DOI:** 10.1038/srep08705

**Published:** 2015-03-03

**Authors:** Xingxing He, Fang Liu, Jingjun Yan, Yunan Zhang, Junwei Yan, Haitao Shang, Qian Dou, Qiu Zhao, Yuhu Song

**Affiliations:** 1 Department of Gastroenterology, Union Hospital, Tongji Medical College, Huazhong University of Science and Technology, Wuhan, P.R. China; 2 Institute of Liver Diseases, Tongji Hospital, Tongji Medical College, Huazhong University of Science and Technology, Wuhan, P.R. China; 3 Institute of Hematology, Union Hospital, Tongji Medical College, Huazhong University of Science and Technology, Wuhan, P.R. China

## Abstract

Reactivation of wild-type p53 (wt-p53) function is an attractive therapeutic approach to p53-defective cancers. An ideal p53-based gene therapy should restore wt-p53 production and reduces mutant p53 transcripts simultaneously. In this study, we described an alternative strategy named as trans-splicing that repaired mutant p53 transcripts in hepatocellular carcinoma (HCC) cells. The plasmids which encoded a pre-trans-splicing molecule (PTM) targeting intron 6 of p53 were constructed and then transfected into HCC cells carrying p53 mutation. Phenotypic changes of HCC cells induced by p53-PTM were analyzed through cell cycle, cell apoptosis and the expression of p53 downstream target genes. Spliceosome mediated RNA trans-splicing (SMaRT) reduced mutant p53 transcripts and produced functional wt-p53 protein after the delivery of p53-PTM plasmids, which resulted in phenotype correction of HCC cells. In tumor xenografts established by p53-mutated HCC cells, adenovirus encoding p53-PTM induced cell cycle arrest and apoptosis and then blocked the growth of tumors in mice. Collectively, our results demonstrated for the first time that mutant p53 transcripts were functionally corrected in p53-defective HCC cells and xenografts using trans-splicing, which indicated the feasibility of using trans-splicing to repair p53 mutation in p53-defective cancers.

Liver cancer in men is the fifth most frequently diagnosed cancer worldwide and the second leading cause of cancer death^1^. An estimated 748,300 new liver cancer cases and 695,900 cancer deaths occurred worldwide in 2008^1^. Half of these cases and deaths were estimated to occur in China^1^. The incidence of liver cancer is increasing worldwide because of the dissemination of hepatitis B and C virus infection. There were an estimated 746,000 deaths from liver cancer in the world in 2012 (http://publications.cancerresearchuk.org/downloads/Product/CS_INFOG_WORLD_MORT.PDF). Epidemiologic and experimental studies have demonstrated that the risk of developing liver cancer was attributable to environmental and genetic factors^2^,^3^,^4^. A multistep process involving accumulation of multiple genetic alterations can occur in malignant transformation of the hepatocytes^3^,^5^. Tumor protein 53 (TP53, well-known as p53) responds to diverse cellular stresses to regulate the expression of target genes, thereby inducing cell cycle arrest, apoptosis, senescence, DNA repair, or changes in metabolism^6^,^7^,^8^. It is now widely acknowledged that p53 mutation is one of the most common genetic events in human cancer^9^. p53 mutations are present in >50% of all human tumors, including hepatocellular carcinoma (HCC)^10^. In HCC, p53 mutation is one of the most common genetic alternations^5^,^11^,^12^. Systematic review and meta-analysis revealed that alterations of p53 were associated with a poor outcome for HCC patients^13^. p53 mutations exhibit a variety of distinct local structural changes and alter thermal stability, which results in the loss of its ability to bind to p53 response elements and trans-activate downstream genes. In addition, mutant p53 proteins are endowed with oncogenic properties such as dominant negative activity and gain-of-function effects, which contributes to the promotion of tumorigenesis^9^,^14^,^15^.

Some strategies for targeting p53 have been developed due to the pivotal role of p53 in tumorigenesis^16^,^17^,^18^. Several gene therapeutic strategies have been employed in the attempt to restore p53 function in p53-defective tumors^16^,^19^,^20^. An ideal approach is the repair of mutant p53 into wt-p53 at molecular level. Firstly, it reduces the expression of mutant p53 which drives tumor progression through dominant negative activity and gain-of-function. Secondly, it induces wt-p53 production which restores wt-p53 activity to suppress the growth of cancer cells. Trans-splicing is a natural process which involves splicing between two separately transcribed mRNAs such that a composite transcript is produced^21^,^22^. Manipulation of this process offers a therapeutic candidate for genetic disorders caused by dominant mutations^21^,^22^,^23^. This property is exploited as an attractive RNA-modification therapy named as spliceosome mediated RNA trans-splicing (SMaRT)^21^,^22^,^23^. SMaRT-mediated repair is achieved by exon replacement and subsequent removal of the defective portion of the target pre-mRNA so that a functional gene product can be transcribed^21^,^22^,^24^. In theory, this strategy simultaneously reduces the production of deleterious mutant p53 protein and restores wt-p53 production. In addition, another advantage of SMaRT over conventional gene therapy is that the corrected genes can be maintained in their native sequence context and are regulated by their endogenous regulatory machinery.

In the context of these considerations, we attempted to correct mutant p53 transcripts in p53-mutated HCC cells using trans-splicing. A pre-trans-splicing molecule (PTM) consisted of trans-splicing domain and 3′ exon that encoded the correct p53 sequence was delivered into HCC cells with p53 mutation. We demonstrated p53-PTM corrected mutant p53 transcripts and restored wt-p53 activities in p53-defective HCC cells, which resulted in the trans-activation of p53-responsive genes and the suppression of the growth of HCC cells *in vitro*. Further study revealed that adenovirus vector expressing p53-PTM blocked the growth of tumor xenografts established by p53-mutated HCC cells.

## Results

### SMaRT-mediated repair of mutant p53 transcripts in transfected cells

**Figure 1:**
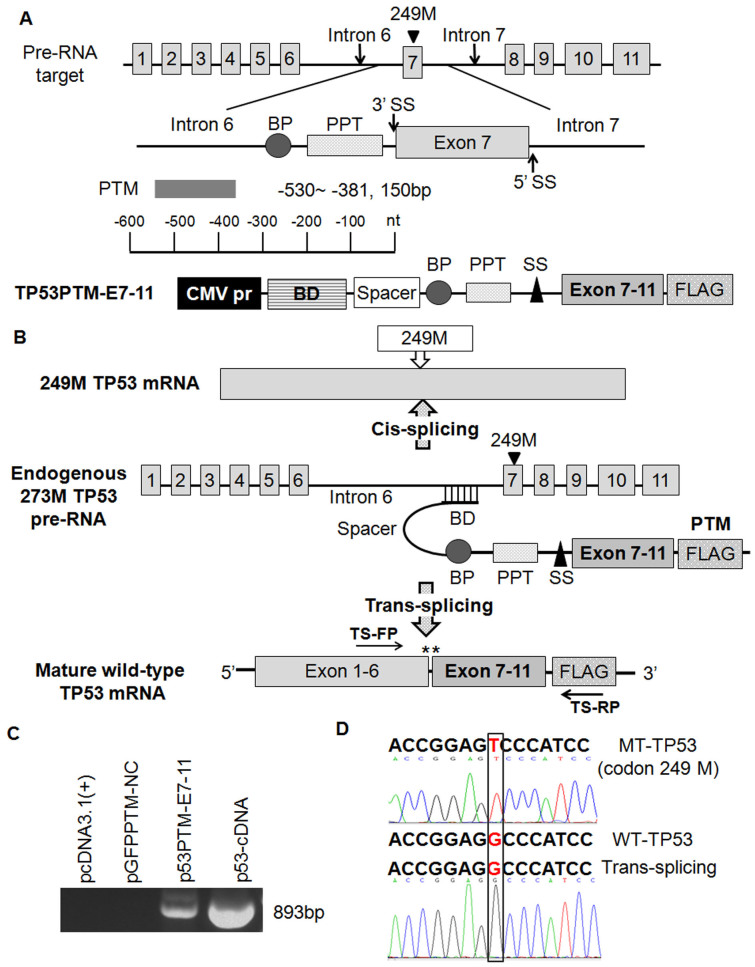
Schematic diagrams of the strategy for trans-splicing-mediated repair of mutant p53 transcripts and evaluation of trans-splicing-mediated repair of p53 mutation in transfected HCC cells. (A). Schematic structure of trans-splicing cassettes and pre-RNA target. p53 pre-trans-splicing molecules contain a binding domain, branch point, polypyrimidine tract, splice site, a coding domain and Flag-tag. (B). Schematic illustration of trans-splicing-mediated repair of mutant p53 transcripts. Cis-splicing of the mutant p53 pre-mRNA yields mutant p53 transcripts in codon 249. Mature wt-p53 transcripts were generated by trans-splicing. Arrowheads indicate the PCR primers used for detection of trans-splicing-generated products. TS-FP: 5′-AGGGCAGCTACGGTT TCCGT-3′, TS-RP: 5′- TACTTGTCATCGTCGTCCTTG-3′. (C). Evaluation of trans-splicing-mediated repair of p53 mutation in PLC/PRF/5 cells by RT-PCR. Trans-spliced p53 RNA yields a reverse transcription-PCR product of 893 bp, no products were observed in PLC/PRF/5 cells transfected with pcDNA3.1(+) or pGFP-PTM. (D). Sequences of RT-PCR products generated from PLC/PRF/5 cells or PLC/PRF/5 cells transfected with p53-PTM. The observed sequences of trans-spliced p53 RNA was identical to that of wt-p53. TP53-S-P is the sequencing primer used for the detection of p53. TP53-S-P: 5′- ATGAGCCGCCTGAGGTTGG-3′. BD, Binding domain; BP, branchpoint; PPT, polypyrimidine tract; SS, splice site.

To test the feasibility of trans-splicing-mediated repair of mutant p53 transcripts *in vitro*, the plasmids encoding p53 trans-splicing expression cassette (Figure 1A) or the controls (Supplementary Figure S1) were delivered into PLC/PRF/5 cells which carried p53 mutation in Exon 7 of p53 cDNA. To confirm p53 trans-splicing in transfected cells, RT-PCR was performed on RNA isolated from the transfected cells using a trans-splicing specific primer set (Figure 1B). RT-PCR analysis detected an expected 893-bp fragments in PLC/PRF/5 cells transfected with p53-PTM (Figure 1C), which was identical to that of p53-cDNA. Moreover, DNA sequencing of the RT-PCR products (Figure 1D) revealed that correct p53 transcripts were generated from splicing events between endogenous target and trans-splicer transcripts, indicating that trans-splicing had occurred correctly with high fidelity in transfected cells. Preferably, the suitable antibodies should be applied to detect repaired p53 protein in transfected cell if possible. Although some antibodies differentiate mutant p53 protein (R175H) from wt-p53^25^; available antibodies do not discriminate mutant p53 protein (R249S) from wt-p53. Therefore, we evaluated the effect of p53-PTM on the expression of p53 protein using p53 antibody which recognizes both mutant forms and wild-type human p53. Western blot result showed that the level of p53 protein was not changed upon the treatment of p53-PTM (Supplementary Fig. S3). Thus, trans-splicing represents a potentially powerful approach for the correction of p53 mutation in HCC cells.

### p53-PTM induced the expression of p53 downstream target genes in transfected cells

**Figure 2:**
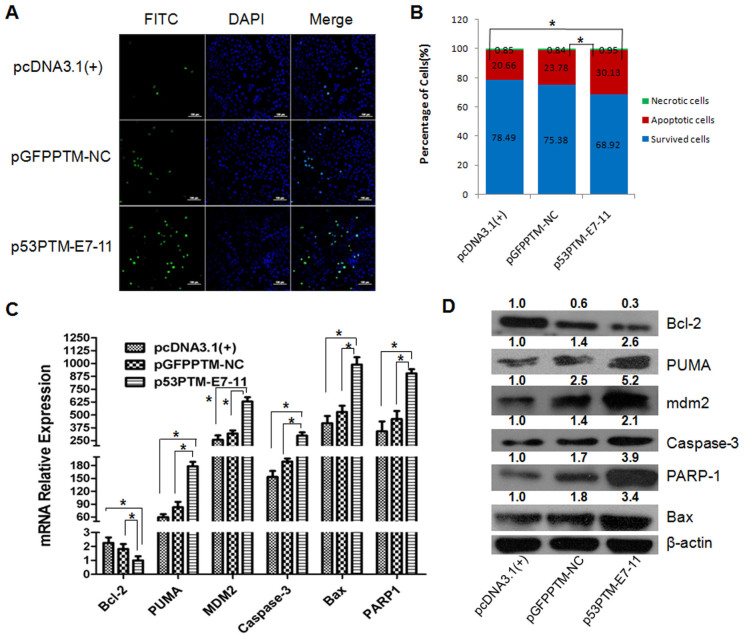
SMaRT-mediated repair of mutant p53 transcripts resulted in the induction of the apoptosis in PLC/PRF/5 cells. (A). SMaRT induced the apoptosis of PLC/PRF/5 cells determined by TUNEL Staining. (B). Cell apoptosis was detected by Annexin-V/propidium iodide combined labeling flow cytometry in PLC/PRF/5 cells. * p < 0.05. (C). mRNA relative expression of p53-responsive apoptotic genes was measured by SYBR Green qRT-PCR. * p < 0.05. (D). Representative Western blot and densitometric quantification of the proteins involved in apoptosis were shown.

The above results confirmed trans-splicing-mediated repair of mutant p53 transcripts in HCC cells which carried p53 mutation in exon 7 of p53. As a transcription factor that both activates and represses a broad range of target genes, wt-p53 acts as a tumor suppressor by inhibiting cell cycle progression, and inducing apoptosis^7^,^15^,^26^. To investigate whether trans-spliced p53 RNAs restore wt-p53 activities in HCC cells, cell apoptosis, cell cycle, cell proliferation, and the expression of p53-dependent downstream genes were determined. Firstly, the apoptosis of transfected PLC/PRF/5 cells was evaluated using terminal deoxynucleotidyl transferase mediated dUTP nick end labeling (TUNEL) staining, annexin V/propidium iodide combined labeling flow cytometry, and the expression of apoptosis-related genes. The result of TUNEL staining (Figure 2A) showed the proportion of TUNEL positive cells increased remarkably in PLC/PRF/5 cells transfected with p53-PTM compared with the controls (p < 0.05). As shown in Figure 2B, flow cytometry using Annexin-V/propidium iodide combined labeling also showed that an increase in the percentage of apoptotic cells was observed in PLC/PRF/5 cells upon the delivery of p53-PTM compared with the controls. PUMA, Bax, PARP-1, caspase-3 are important indicators of cell apoptosis. As shown in Figure 2 C and D, increased expression of caspase-3, PUMA and PARP was observed in PLC/PRF/5 cells transfected with p53PTM-E7-11. Moreover, p53-PTM suppressed the expression of the apoptosis-suppressing gene Bcl-2, and simultaneously stimulated the expression of Bax which encodes a dominant inhibitor of the Bcl-2 protein. Mdm2, a downstream molecule of p53, has been well characterized as a negative regulator. Mdm2 was also been induced in PLC/PRF/5 cells transfected with p53 PTM (Figure 2C and D).

**Figure 3:**
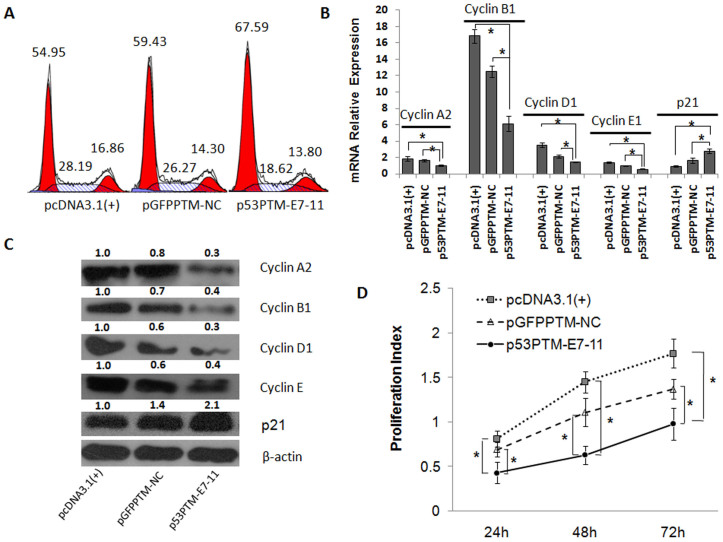
The effect of p53-PTM on the proliferation and cell cycle of PLC/PRF/5 cells. (A). Flow cytometry combined with propidium iodide staining demonstrated that SMaRT induced cell cycle arrest in PLC/PRF/5 cells upon the treatment of p53-PTM. Numbers over each histogram indicated the percentage of the cells in G0-G1, S, and G2-M phase. (B). mRNA relative expression of cyclins and p21 was measured by SYBR Green qRT-PCR. *p < 0.05. (C). Representative Western blot and densitometric quantification of the cyclins and p21 were shown. (D). Proliferation assay determined proliferative activity of PLC/PRF/5 cells after transfection of p53-PTM or the controls. * p < 0.05.

Then, the effect of p53-PTM on cell cycle was evaluated through analyzing the distribution of cell cycle and the expression of regulatory genes for cell cycle. As shown in Figure 3A, flow cytometry analysis demonstrated an accumulation of cells in the G0-G1 phase following the delivery of the plasmid carrying p53-PTM into PLC/PRF/5 cells. Of the many genes involved in cell cycle control, cyclins control the progression of cells through the cell cycle by activating cyclin-dependent kinase (Cdk) enzymes. Partial repair of mutant p53 transcripts by SMaRT resulted in down-regulation of cyclins, which was demonstrated by quantitative RT-PCR analysis (Figure 3B) and immunoblot results (Fig 3C). p21, a regulator of cell cycle progression, is controlled by p53. Induction of p21 was observed in PLC/PRF/5 cells transfected with PTM-p53 compared with the controls (Figure 3B and C). Finally, proliferative activity was inhibited in PLC/PRF/5 cells after the transfection of p53-PTM compared with the controls (Figure 3D).

To further confirm whether p53-PTM induce p53-dependent downstream genes in cancer cells, the promoter activities of p53 downstream genes were determined using luciferase assay. As shown in Supplementary Figure S4, p53-PTM-E7-11 trans-activated the exogenously expressed luciferase gene driven by PUMA and p21 promoter. In addition, the results showed that the promoter activities of cyclin A, B, D and E and Bcl-2 were downregulated in PLC/PRF/5 cells treated with p53-PTM (Supplementary Figure S4).

Therefore, *in vitro* data demonstrated p53-PTM suppressed the growth of PLC/PRF/5 cells significantly compared with the negative control (pcDNA3.1), which resulted from an induction of wt-p53 production and a reduction in mutant p53 transcripts. Simultaneously, the above protocols revealed a weak induction of apoptosis, cell-cycle arrest in pGFPPTM-NC transfected cancer cells compared with the negative control (pcDNA3.1). It might be caused by a reduction in mutant p53 transcripts.

### The effect of SMaRT on the growth of HCC xenograft tumors in nude mice

**Figure 4:**
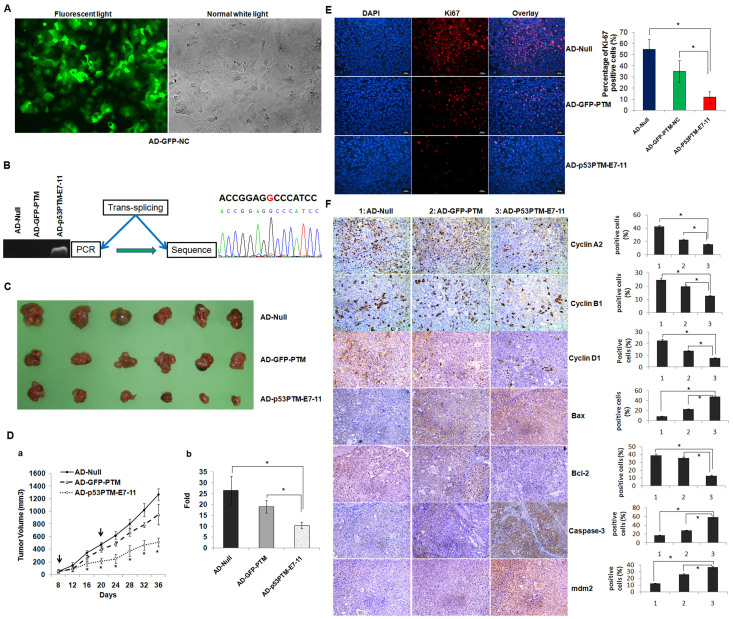
Adenovirus vectors expressing p53-PTM inhibited the growth of xenograft tumors developed by PLC/PRF/5 cells in nude mice. (A). The result showed efficient adenoviral transduction of PLC/PRF/5 cells as indicated by eGFP expression using a fluorescence microscope, 2 days following adenovirus vector administration. The black and white pictures showed the cells in the same field under normal white light. (B). Detection of trans-spliced p53 RNA in xenograft tumors. RT-PCR results showed that trans-spliced p53 RNAs were generated in PLC/PRF/5 cells infected with adenovirus vectors expressing p53-PTM (AD-p53PTM-E7-11). DNA sequencing of the RT-PCR products of trans-spliced p53 RNA confirmed trans-splicing-mediated repair of mutant p53 transcripts in the infected cells with high fidelity. (C). Effects of AD-p53PTM on the growth of pre-established PLC/PRF/5 xenografts at a gross morphology level. (D). Adenovirus vectors expressing p53-PTM inhibited the growth of xenograft tumors developed by PLC/PRF/5 cells in nude mice. The inhibitory effect of AD-p53PTM on the growth of xenograft tumors was revealed by the tumor growth curves (a) and the average volume fold increase of tumors at the sacrifice with respect to the first measurements (b). *p < 0.05. (E). Cell proliferation was assessed by immnofluorescence staining of Ki67. Representative immunofluorescence staining and the percentage of ki67-positive cells were shown. *p < 0.05. (F). The expression of the proteins involved in cell cycle and apoptosis was evaluated by immunohistochemical staining of the sections from the xenograft tumors. Representative immunohistochemical staining and percentages of cells staining positive for cyclins, Bax, Bcl-2, caspase3 and mdm2 were shown. Positive cells were counted in tumor tissues and presented as the mean ± SD (4 random fields per section and three sections per tumor). 1: AD-Null, 2: AD-GFP-PTM, 3: AD-p53PTM-E7-11. * p < 0.05.

To evaluate trans-splicing-mediated repair of mutant p53 transcripts *in vivo*, adenovirus vectors carrying trans-splicing cassette of p53 were successfully prepared and their high infection efficiency was obtained in PLC/PRF/5 cells as indicated by eGFP expression (Figure 4A). Adenovirus vectors were administered directly into xenograft tumors developed by PLC/PRF/5 cells. 48 hours later, total RNA extracted from tumors was subjected to RT-PCR analysis for the detection of trans-spliced p53 RNA. As expected, an anticipated product of 893 bp was detected in RNA from tumors injected with adenovirus encoding p53-PTM, and no amplification was detected from tumors injected with AD-GFP-PTM and AD-Null (Figure 4B). RT-PCR product was sequenced and the result showed point mutation of p53 transcripts was repaired by SMaRT (Figure 4B). Based on the crucial role of p53 in the pathogenesis of HCC, we further pursued the ability of SMaRT against p53 in suppressing the growth of xenograft tumors. Adenovirus vectors expressing p53-PTM and the controls were injected directly into the tumors, and tumor size was monitored over time. Although the tumors grew progressively in all groups during the course of the experiment, tumors in p53 PTM-treated mice were significantly smaller than those in control mice reflected by the gross morphology (Figure 4C) and growth curves (Figure 4D). The growth curves of treated tumors became divergent on day 16, and the average fold increase of tumor volumes at the sacrifice with respect to the first measurements in AD-p53PTM-treated group was less than that in controls (Figure 4D).

### The effect of p53-PTM on the proliferation, the expression of Cyclins, Bax, Bcl-2, caspase-3 and mdm2 in xenograft tumors

Proliferative phenotypes are fundamental components of malignant diseases. In contrast with AD-Null or AD-GFP-PTM, the tumor cells in AD-p53PTM-E7-11 group manifested a remarkable decrease in immunofluorescence of proliferative biomarker ki67 (Figure 4E). Bcl-2 family members, caspase-3, cyclins are important indicators of cell growth arrest and apoptosis. In tumor tissues examined by immunohistochemical staining, administration of AD-p53PTM-E7-11 resulted in remarkable up-regulation of caspase-3 and Bax, accompanied with decreased expression of Bcl-2 and cyclin A, B, D (Figure 4F). Simultaneously, tumors in mice treated with AD-p53PTM-E7-11 showed significant induction of p53-responsive molecule mdm2 compared with the controls. In addition, consistent with the effect of GFP-PTM on cell cycle and apoptosis in transfected cells *in vitro*, adenovirus enconding GFP-PTM showed week induction of cell-cycle arrest and cell apoptosis compared with the negative control (AD-Null) (Figure 4F).

## Discussion

Rescuing the function of mutant p53 in cancer cells is an attractive cancer therapeutic strategy. In this study, a RNA-based strategy named as SMaRT has been designed to amend mutant p53 transcripts in HCC cells carrying mutant p53 transcripts. We demonstrated a plasmid carrying p53-PTM mediated the correction of mutant p53 transcripts in p53-defective HCC cells. The reactivation of wt-p53 in PLC/PRF/5 cells led to the transactivation of p53-dependent target genes, and then induced cell cycle arrest and cell apoptosis *in vitro*. Human tumor xenografts have been widely used as predictive preclinical models for anticancer drug in humans. Therefore, HCC xenograft is used as a tumor model for evaluating the feasibility of trans-splicing. After intratumoral injection of adenovirus vector encoding p53-PTM, SMaRT reduced the growth of xenograft tumors developed by PLC/PRF/5 cells. Thus, it indicated that endogenous p53 mutant transcripts were partially corrected using SMaRT *in vivo*. Intra-tumor delivery is of limited value for most tumor types; however, local therapeutic approaches such as local ablation with radiofrequency or percutaneous ethanol injection, transcatheter arterial chemoembolization were also recommended in HCC clinical practice guideline. HCC xenografts and orthotopic HCC tumor model share similar characteristic for intratumor administration of anticancer drug. These indicate that intratumor delivery of vectors expressing p53-PTM is feasible for the treatment of p53-mutated HCC.

Mutation or functional inactivation of p53 is one of the most common genetic events in human cancer, including HCC. Most p53 mutations are missense point mutations that preferentially localized in the DNA binding domain^9^,^15^. A single missense mutation at codon 249 of the p53 gene has been identified as one of the “hotspot” mutation^9^,^15^. Therefore, PLC/PRF/5 cells containing mutation in codon 249 were selected as cell model for the detection of SMaRT-mediated correction of mutant p53 transcript. p53 mutations in the DNA binding domain result in the disruption of DNA binding and transcriptional trans-activation of p53 target genes, which abrogates p53 tumor-suppressive functions. In addition, mutant p53 protein gains additional oncogenic functions that endow cells with growth and survival advantages. A number of strategies based on the reactivation of wt-p53 function in p53-defective cancer cells have been developed^18^,^27^. Numerous studies have demonstrated that small synthetic molecules and peptides reversed phenotype of cancer cells *in vitro* and *in vivo* by reconstituting p53 tumor suppressor functions^18^,^27^. While, the chemicals had potential toxicity which might limit their application in humans. Their molecular mechanism underlying restoration of p53 function should be pursued, and the application of the chemicals in clinical practice should be evaluated in further study. In theory, restoring mutant p53 function and/or enhancing wt-p53 by genetic means is a viable and attractive approach for developing cancer therapeutics. The efficacy of wt-p53 administration has been confirmed in preclinical and clinical models^28^,^29^,^30^. However, over-expression of p53 might be found in mice tissues after the administration of wt-p53 and increased p53 activity in mice model displayed the phenotype of accelerated and pronounced aging^31^,^32^,^33^. Mutant p53 proteins are unusually stable and accumulate to high levels in cancer cells, which exert gain-of-function in the promotion of carcinogenesis^9^,^14^,^15^. Therefore, the strategy of wt-p53 administration did not get a rid of detrimental mutant p53 transcripts. Alternative approach is knockdown of endogenous p53 mutant transcripts in cancer cells using siRNAs which inhibited tumor growth^34^,^35^,^36^. However, absence of wt-p53 expression in cancer cells contributed to the development of various tumor types^32^. Therefore, an ideal strategy for p53-mutated cancer cells is the repair of mutant p53 into wt-p53 in molecular level, which can simultaneously reduce mutant p53 expression and restore wt-p53 expression in cancer cells. Trans-splicing ribozymes had been utilized to repair mutant p53 transcripts with high fidelity and specificity in cancer cells^37^,^38^. Trans-splicing ribozymes-mediate repair of mutant p53 resulted in the production of functional p53 and the reduction of mutant p53 expression^37^,^38^. Unfortunately, ribozyme stability and efficiency are the main hurdles to applying the ribozyme to the clinic^22^,^24^,^37^,^38^. SMaRT can result in the replacement of defective target pre-mRNA via a correct version of the segment. This technology has been successfully applied in phenotypic correction of genetic defects, including cystic fibrosis (CF), haemophilia A, and X-linked immunodeficiency^39^,^40^,^41^,^42^. Our studies have clearly demonstrated for the first time that partial correction of mutant p53 transcripts was achieved using SMaRT. All p53 isoforms share same structure in intron 6 and exon 7^43^, which results in successful correction of all p53 isoforms containing p53 mutation in exon 7 using p53PTM-E7-11. The trans-spliced p53 transcripts induced apoptosis and cell cycle arrest in transfected cells and xenograft tumors developed by PLC/PRF/5 cells. As a RNA-based strategy, SMaRT has several advantages over conventional gene therapy. Trans splicing-mediated revision of mutant p53 transcripts can simultaneously reduce mutant p53 transcripts and restore wt-p53 production, which conventional gene replacement does not address. As the gene is repaired rather than introduced, the spatial and temporal expression of the gene should be controlled by endogenous regulation. Coordinated expression of the p53 is apparently critical for proper homeostasis of cells growth, and incorrect p53 expression in mice model can lead to phenotypic aberrations^33^,^44^. The corrected p53 genes by SMaRT can be maintained in their native sequence context and are regulated by their endogenous regulatory machinery. In addition, the protein expression of p53 was not changed in HCC cells or normal hepatic cells expressing wt-p53 upon the treatment of p53-PTM (Supplementary Fig S3). Thus, trans-splicing-mediated repair of mutant p53 transcripts takes place in p53-defective cancer cells, and undesirable effects of SMaRT on normal cells would be avoided.

PLC/PRF/5 cell line also contains other gene mutations such as CDKN2A (R112G). CDKN2A binds to mdm2 and inhibits the oncogenic action of mdm2 by blocking mdm2-induced degradation of p53 and enhancing p53-dependent transactivation and apoptosis^45^. However, Weber JD et al demonstrated that CDKN2A could also exert its tumor suppressive functions independently of the mdm2-p53 axis^46^. In addition, recent studies demonstrated that CDKN2A mutation did not occur frequently in human HCC tissues^47^,^48^. So, CDKN2A mutation may impair the therapeutic effects of p53-PTM on xenograft tumors created by PLC/PRF/5. However, our study focused on the repair of mutant p53 transcripts using trans-splicing in p53-mutated HCC cells. Therefore, the effects of p53-PTM on the function of other gene mutations were not investigated.

Of course, SMaRT has its limitations in the repair of p53 mutation. One of the major limitations is that a single PTM would not be able to address all mutations across a defective gene. Low efficiency would be a major potential disadvantage for the application of trans-splicing. Although p53-PTM suppressed the growth of p53-defective HCC cells *in vitro* and *in vivo* significantly, improvements in PTM design and delivery system which will increase their efficacy should be made in further study.

Mutations of p53 are one of the most frequent molecular events in the tumorigenesis of HCC, and play an important role in the pathogenesis of HCC. In conclusion, our study demonstrated that SMaRT was successfully applied to correct mutant p53 transcripts in HCC cells carrying p53 mutation, which resulted in restoration of wt-p53 activity and phenotypic correction of HCC cells. These revealed that SMaRT would be developed as a good therapeutic candidate for p53-defective HCC.

## Methods

### Production of recombinant adenovirus and animal experiment

Trans-splicing expression cassettes were cloned into shuttle plasmid of AdMax™ system with Cre-lox (Microbix Biosystems Inc., Ontario, Canada). The recombinant adenoviruses were produced and purified. Six to eight-week-old Nu/Nu mice obtained from Beijing HFK Bioscience Co. LTD (Beijing, China) were inoculated subcutaneously with PLC/PRF/5 cells to generate tumor xenografts, and then adenovirus vectors were administered directly into the tumors. At the end of the experiment, the mice were sacrificed at day 36 post-inoculations and the tumors were collected. Immunohistochemistry and immunofluoresence were performed on tumor tissues as described previously^50^,^51^.

### Ethic statement

The study was approved by the local research ethics committee at the Tongji Hospital of Huazhong University of Science and Technology. The methods used in this study were carried out in accordance with the approved guidelines.

### Statistical analysis

All data are expressed as mean ± standard error from 3 separate experiments performed in triplicate except otherwise noted. The differences between groups were analyzed by Student's t test and *P* < 0.05 was considered to be statistically significant.

## Author Contributions

X.X.H., F.L. and Y.H.S. designed the research; X.X.H., F.L., J.J.Y., Y.N.Z. and J.W.Y. performed the research; H.T.S., Q.D. and Q.Z. contributed new reagents and analyzed the data; Y.H.S. and X.X.H. wrote the paper.

## Supplementary Material

Supplementary Information Supplementary data
