# Supplementary material for: Trans-splicing repair of mutant p53 suppresses the growth of hepatocellular carcinoma cells in vitro and in vivo
Source: Sci Rep. 2015 Mar 3;5:8705. doi: 10.1038/srep08705 (PMC4346796; doi:10.1038/srep08705)
Supplement: Supplementary Information — Supplementary data [file srep08705-s1.pdf]

Supplemental Data

Trans-splicing repair of mutant p53 suppresses the growth of hepatocellular carcinoma cells *in vitro* and *in vivo*

Xingxing He, Fang Liu, Jingjun Yan, Yunan Zhang, Junwei Yan, Haitao Shang, Qian Dou, Qiu Zhao, Yuhu Song

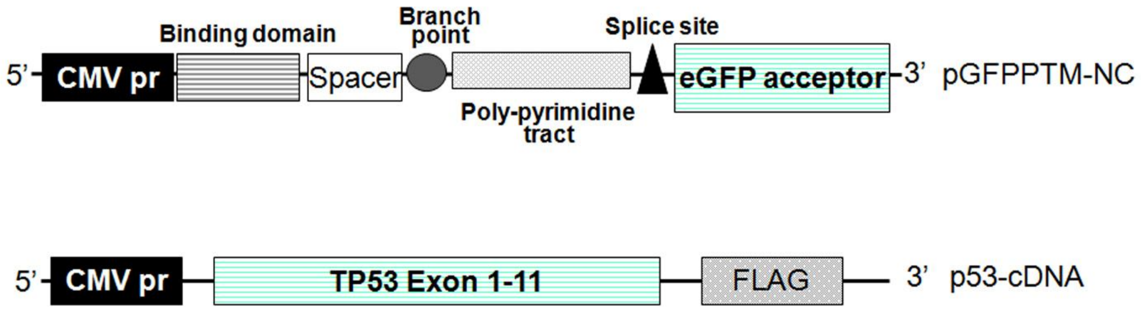

Figure S1 Schematic illustration of the plasmid constructs pGFPPTM-NC and p53-cDNA, which are used as the control for p53PTM-E7-11. pGFPPTM-NC contained CMV promoter, binding domain, spacer, branch point, poly-pyrimidine tract, splice acceptor site, eGFP acceptor (GenBank accession # U55763.1 nt 995-1410) and Flag-tag. GFP-PTMs pair specifically with intron 6 of p53 gene, and then replace mutant piece of p53 mRNA with GFP acceptor to produce a hybrid transcripts of exons 1-6 of p53 mRNA and GFP acceptor. The plasmid of p53 cDNA contained CMV promoter, p53 cDNA (GenBank accession # NM_000546 nt 198-1379) and Flag-Tag, which was used as a positive control.

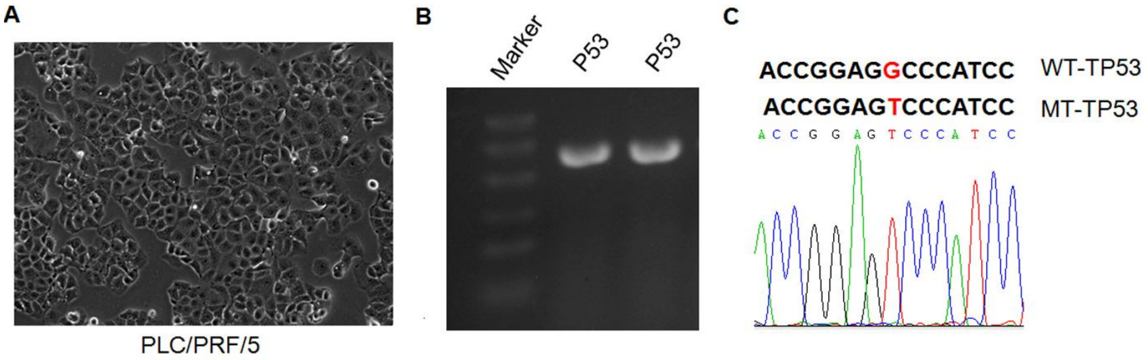

Figure S2 Detection of a mutation in codon 249 of p53 gene in human hepatoma cell line PLC/PRF/5.

A. The micrographs of human hepatoma cell line PLC/PRF/5.

B. Part of TP53 cDNA containing a mutation in codon 249 was obtained by RT-PCR. RT-PCR was carried out on total RNAs isolated from human hepatoma cell line PLC/PRF/5. The primers used for RT-PCR (TP53-FP and TP53-RP) were listed in Supplementary Table1.

C. DNA sequencing results of the RT-PCR products confirmed a mutation in codon 249 of the p53 gene in PLC/PRF/5 cells.

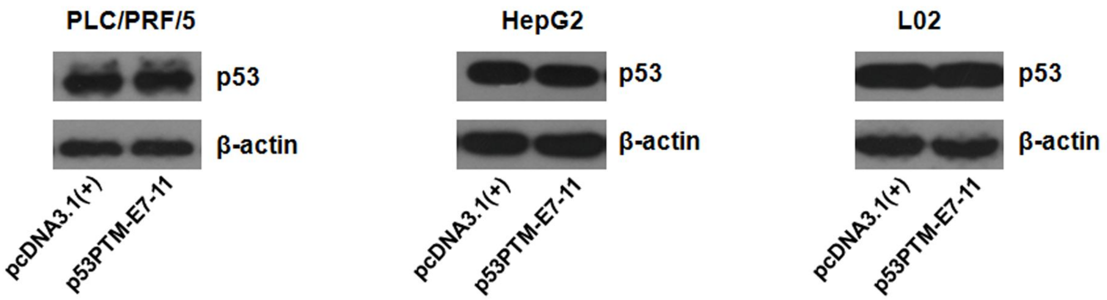

Figure S3 The effects of p53PTM-E7-11 on p53 protein expression in PLC/PRF/5, HepG2 and L02 cells. HepG2 is a HCC cell line expressing wild-type p53; L02 is a normal hepatic cell line that expresses wild-type p53. The results showed that the protein expression level of p53 was not obviously changed in PLC/PRF/5, HepG2 or L02 cells upon the treatment of p53PTM-E7-11.

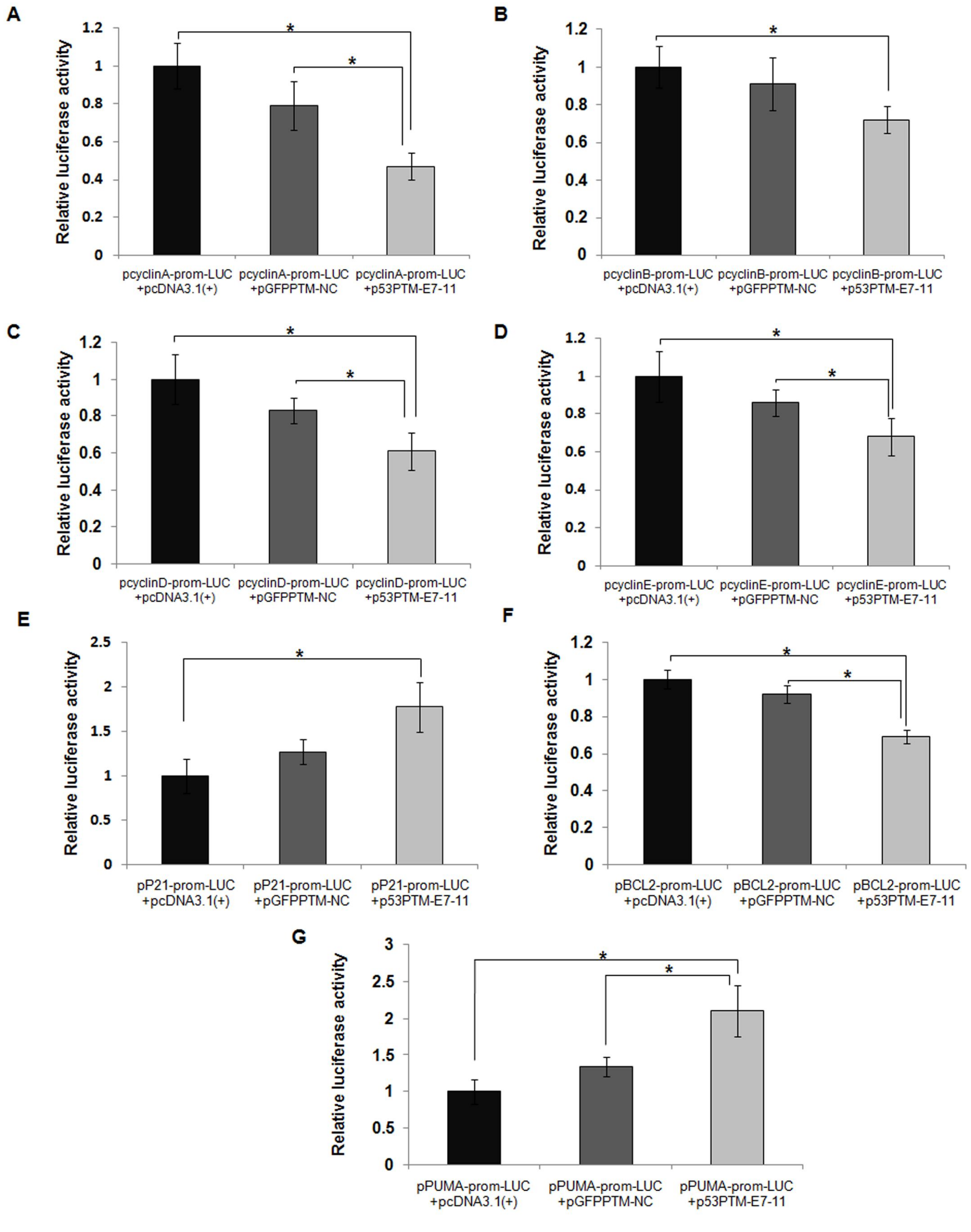

Figure S4. The effect of p53PTM-E7-11 on the promoter activity of p53 downstream genes. PLC/PRF/5 cells were co-tranefected with 0.1 μg p53PTM-E7-11 or the controls, 0.1 μg luciferase reporter constructs, and 0.01μg of pMIR-REPORTTM β-gal plasmid

as an internal control for transfection efficiency. At 48 h after transfection, luciferase and β--galactosidase activities were measured. Relative activity of luciferase was standardized to β-gal signal and the relative activity of luciferase from control group (pCDNA3.1) is designated as 1. * $P < 0.05$.

A. Repression of cyclin A promoter in PLC/PRF/5 cells transfected with p53PTM-E7-11.

B. Repression of cyclin B promoter in PLC/PRF/5 cells transfected with p53PTM-E7-11.

C. Repression of cyclin D promoter in PLC/PRF/5 cells transfected with p53PTM-E7-11.

D. Repression of cyclin E promoter in PLC/PRF/5 cells transfected with p53PTM-E7-11.

E. Induction of p21 promoter in PLC/PRF/5 cells transfected with p53PTM-E7-11.

F. Repression of Bcl-2 promoter in PLC/PRF/5 cells transfected with p53PTM-E7-11.

G. Induction of PUMA promoter in PLC/PRF/5 cells transfected with p53PTM-E7-11.

Supplementary Methods

Cell culture

Human hepatocellulae cancer cells HepG2 (ATCC HB-8065) , PLC/PRF/5 (ATCC CRL8024) were cultured in DMEM, supplemented with 10% fetal bovine serum, 100 units/mL of penicillin, and 100 µg/mL of streptomycin at 37°C in a humidified atmosphere of 5% CO2. Normal hepatocyte L02 cells were cultured in 1640 supplemented with 10% fetal bovine serum. PLC/PRF/5 contains p53 mutation in codon 249 (R249S). HepG2 is a HCC cell line expressing wild-type p53; L02 is a normal hepatic cell line that expresses wild-type p53.

Trans-splicing cassette

As shown in **Figure 1**, the plasmid carrying p53 pre-trans-splicing molecule included CMV promoter, a binding domain, the splice elements, a coding domain and and Flag-tag. The spacer was comprised of a *de novo* sequence (5'-GCGGCCGCCCGAATAA GTGATTGA TTGAG TTT-3'), that is non-homologous to any murine or human sequence. This was followed by the artificial branch point and polypyrimidine tract and splice acceptor sequence (5'- TACTAACTGAT ATCTCT TCTTTTTTTTTT CCGGA AAACAG-3') as described previously [1-3]. The sequence of FLAG-tag is 5' GAC TAC AAG GAC GAC GAC GAC AAG -3'. In this study, the plasmids (pGFPPTM-NC and p53-cDNA) encoding GFP pre-trans-splicing molecule or p53 cDNA were constructed as the controls of p53PTM-E7-11 (**Supplementary Figure S1**). All the plasmids were based on a backbone derived from the pcDNA3.1(+) vector (Invitrogen, Carlsbad, CA). For pGFP-PTM (Figure S1), the only difference between pGFPPTM-NC and p53PTM-E7-11 was that eGFP acceptor (GenBank accession # U55763.1 nt 995-1410) in pGFP-PTM replaced exons 7-11 of p53 cDNA in p53PTM-E7-11. For the plasmid expressing p53 cDNA, p53 cDNA were obtained by RT-PCR and then cloned in to pcDNA3.1(+) vector backbone. As shown in Figure S1, Flag-tag followed by p53 cDNA was added into the vector. All the primers used for plasmids construction were listed in **Supplementary Table S1**.

RT-PCR and Real-time PCR

Total RNA (5 μg) were extracted from the cells or the tissues and reverse transcribed into cDNA using PrimeScript RT reagent Kit (DRR037S; Takara, Dalian, China). The cDNAs were used as templates for subsequent PCR using the Platinum PCR SuperMix High Fidelity (Invitrogen, Carlsbad, California, USA). To detect trans-spliced p53 transcripts in transfected or infected cells, PCR was done using the primers specific to exon 4 and Flag-tag (**Supplementary Table S1**). The PCR products were purified by gel extraction kit, and sequenced. Real-time PCR was performed using SYBR Green PCR Kit (Applied Biosystems, Foster City, CA). Data analysis was performed using the 2△△Ct method. Each sample was tested in triplicate. Threshold values were determined for each sample and average and standard error values were calculated. mRNA levels were normalized to the level of GAPDH in the same sample. Primers used were listed in the **Supplementary Table S2**.

TUNEL Staining

PLC/PRF/5 cells grown on chamber slides were transfected with p53-PTM or the controls. After 48 hours, cells were fixed using 2% paraformaldehyde and TUNEL staining was performed by terminal deoxynucleotidyl transferase-mediated nick end labeling staining (In Situ Cell Death Detection kit, Fluorescein, Roche Diagnostics, Mannheim, Germany). Nuclei were stained with 4,6-diamidino-2-phenylindole (DAPI). Then, two independent investigators, blinded to the treatment, evaluated the relative number of apoptotic cells per well by counting 4 randomly selected high-power fields.

Western blot

Lysates from the cells or the tissues were collected using RIPA buffer (Sigma R0278, St Louis, MO, USA). The lysates were resolved over SDS-polyacrylamide gels, and blotted onto polyvinylidene difluoride membrane. The specimens were immunoblotted with primary antibodies, then with peroxidase-conjugated secondary antibodies. Immmunoreactive bands were visualized with ECL detection kit (Thermo 34077, Rockford, IL, USA). The following antibodies were used: anti-Cyclin A2 (1540-1, Epitomics, Burlingame, CA, USA), anti-p53 (10442-1-AP, Proteintech), anti-Cyclin

B1(1495-1, Epitomics), anti-Cyclin D1(1677-1, Epitomics), anti-Cyclin E (4129, Cell Signaling Technology, Beverly, MA, USA), anti-Bcl-2 (1017-1, Epitomics), anti-PUMA (1652-1, Epitomics), anti-mdm2 (556353, BD PharMingen, San Diego, CA,USA), anti-Caspase-3 (1087-1, Epitomics), anti-p21(2990-1, Epitomics), anti-PARP-1 (1072-1, Epitomics), anti-Bax (1063-1, Epitomics), anti-β-actin (60008-1-Ig, Protein Tech, Chicago, IL, USA), goat anti-mouse IgG-HRP (sc-2005, Santa Cruz Biotechnology, Dallas, Texas) and goat anti-rabbit IgG-HRP(sc-2004, Santa Cruz Biotechnology).

Cell cycle and cell apoptosis assay

Cell cycle was analyzed using propidium iodide DNA staining. In brief, transfected cells were harvested, and fixed with 70% ethanol. After the incubation with ribonuclease, the specimens were stained with propidium iodide. The results were evaluated by flow cytometry. Cell apoptosis was detected using Annexin V FITC apoptosis detection Kit (Keygentec, Nanjing, China). Briefly, the transfected cells were suspended in binding buffer. After the cells were incubated with the solution of Annexin V-FITC and propidium iodide, the results were analyzed by a flow cytometer (FACS Calibur, Becton Dickinson, USA).

Cell proliferation

Cell proliferation was evaluated by CellTiter 96 aqueous one solution cell proliferation assay kit (Promega, USA). In brief, the transfected cells were incubated with the CellTiter One Solution Reagent, and the absorbance was measured at 490 nm.

Luciferase assay

PLC/PRF/5 cells seeded in 96-well plates were transiently transfected with 0.1 μg p53PTM-E7-11 or the control plasmids, 0.1 μg luciferase reporter constructs, and 0.01μg of pMIR-REPORTTM β-gal plasmid as an internal control for transfection efficiency. At 48 h after transfection, luciferase and β--galactosidase activities were measured using the Dual-Light System (Applied Biosystems) as we have described before [4]. The following reporter constructs of promoter were used in transfection experiments: pcyclinA-prom-LUC (Constructed by ourselves, as described by Henglein et al) [5],

pcyclinB-prom-LUC (provided by Prof Giulia Piaggio, Experimental Oncology Department, Istituto Regina Elena, Italy) [6], pcyclin D-prom-LUC (32727, Addgene) [7] , pcyclin E-prom-LUC (8458, Addgene) [8], pP21/WAF1-prom-LUC (16451, Addgene [9], pBCL2-prom-LUC (15393, Addgene) [10], pPUMA-prom-LUC (16591, Addgene) [11].

Production of recombinant adenovirus and cell infection

Trans-splicing expression cassettes containing one binding domain, the splice elements, a coding domain (exons 7-11 of p53 cDNA) and Flag-tag were cloned into shuttle plasmid of AdMax™ system with Cre-lox (Microbix Biosystems Inc., Ontario, Canada). The recombinant adenoviruses packaging p53-PTM and the controls were produced in 293 cells and then purified through Adeno-X™ Virus Purification Kit (BD Biosciences, Clontech). PLC/PRF/5 cells were infected with adenovirus vector, and then collected for further study at 48 hours post-transfection.

Animal experiment

Six to eight-week-old Nu/Nu mice obtained from Beijing HFK Bioscience Co. LTD (Beijing, China) were inoculated subcutaneously with 2×10^6 PLC/PRF/5 cells. Tumors of comparable size were established at 8 days post-injection, and then 2×10^8 TU adenovirus vectors per mouse were administered directly into the tumor twice (Day 8, 20) in a 100 μl volume. The transplanted nude mice were randomly divided into 3 groups (n = 6 each). Tumor size was measured by calipers every 4 days and tumor volume was calculated using the formula (length × width × width/2). The tumor tissues were harvested and processed for the analysis of immunohistochemistry and inmmunofluoresence. The protocol of animal treatment used in this study was approved by the institutional animal care and use committee.

Statistical assessment

All data are expressed as mean ± standard error from 3 separate experiments performed in triplicate except otherwise noted. Differences between means were analyzed by Student's t-test and $P < 0.05$ was considered to be statistically significant.

Table S1 Primers used for plasmids construct, the detection of trans-splicing and the amplification of p53 cDNA.

**Primer names**	**Purpose**	**Sequences (5' to 3')**
TP53-FP	Amplification of p53 part cDNA used for the detection of p53 mutation	CCC CAC CAT GAG CGC TGC TC
TP53-RP	Amplification of p53 part cDNA used for the detection of p53 mutation	CGC TCA CGC CCA CGG ATC TG
TP53-S-P	Sequencing primer used for the detection of p53 mutation	ATG AGC CGC CTG AGG TTG G
BD-FP	Amplification of binding domain	CGC GGATCC GCC CTT AGC CTC TGT AAG CTT CAG
BD-RP	Amplification of binding domain	CCG GAATTC AGG GTG GTT GTC AGT GGC CCT
p53-E7-11-FP	Amplification of exon 7-11 of p53	ATAAGAAT GCGGCCGC TCT TCT TTT TTT TGC AGG TTG GCT CTG ACT GT
p53-E7-11-RP	Amplification of exon 7-11 of p53	CCGCTCGAG CTA **CTTGTCATCGTCGTCCTTGTAGTC** GTC TGA GTC AGG CCCT
p53-cDNA-FP	Amplification of p53 cDNA	CGC GGATCC GCC ACC ATG GAG GAG CCG CAG
p53-cDNA-RP	Amplification of p53 cDNA	CCGCTCGAG CTA **CTTGTCATCGTCGTCCTTGTAGTC** GTC TGA GTC AGG CCC TT
TS-FP	Detection of trans-spliced p53 RNA	AGG GCA GCT ACG GTT TCC GT
TS-RP	Detection of trans-spliced p53 RNA	TAC TTG TCA TCG TCG TCC TTG

FP, forward primer; RP, reverse primer. BamHI:GGATCC; EcoRI:GAATTC; NotI:GCGGCCGC; XhoI:CTCGAG;

FLAG: **CTT GTC ATC GTC GTC CTT GTA GTC.**

Table S2. Primers used in SYBR Green qRT-PCR.

Primer names	Sequences (5' to 3')
**cyclin A2-FP**	CAGACGGCGCTCCAAGAGGA
**cyclin A2-RP**	AGGGGTGCAACCCGTCTCG
**cyclin B1-FP**	TCCGAGTCACCAGGAACTCGAAAA
**cyclin B1-RP**	AGTCCGGGCTTGGAGGTTGC
**cyclin D1-FP**	GCGTACCCCGATGCCAACCT
**cyclin D1-RP**	CCTCGCAGACCTCCAGCATCC
**cyclin E1-FP**	CAGGGGCGTCGCTGATGAAGA
**cyclin E1-RP**	TGCTCGGGCTTTGTCCAGCAA
**BCL2-FP**	CGGAGGCTGGGATGCCTTTGT
**BCL2-RP**	AGCTCCCACCAGGGCCAAAC
**p21-FP**	CACTCAGAGGAGGCGCCATGT
**p21-RP**	GCCCGCCATTAGCGCATCAC
**BAX-FP**	GCAAACTGGTGCTCAAGGCCC
**BAX-RP**	TCTCACCCAACCACCCTGGTCT
**PUMA-FP**	TGGGACTCCTGCCCTTAC
**PUMA-RP**	GGCTGGGAGTCCAGTATG
**MDM2-FP**	GCGCCCCGTGAAGGAAACTG
**MDM2-RP**	TGCACATTTGCCTGCTCCTCAC
**CASP3-FP**	GGCGGTTGTAGAAGAGTTTCGTGA
**CASP3-RP**	ACCGAAAACCAGAGCGCCGA
**PARP1-FP**	GATTGCTGTGGCACGGGTCC
**PARP1-RP**	TAGCCTGTCACGGGCGCTTC
**GAPDH-FP**	CCCCAGCAAGAGCACAAGAG
**GAPDH-RP**	GCACAGGGTACTTTATTGATGGTAC

FP, forward primer; RP, reverse primer

References:

1 Liu, X. *et al.* Partial correction of endogenous DeltaF508 CFTR in human cystic fibrosis airway epithelia by spliceosome-mediated RNA trans-splicing. *Nat Biotechnol* **20**, 47-52 (2002).

2 Chao, H. *et al.* Phenotype correction of hemophilia A mice by spliceosome-mediated RNA trans-splicing. *Nat Med* **9**, 1015-1019 (2003).

3 Tahara, M. *et al.* Trans-splicing repair of CD40 ligand deficiency results in naturally regulated correction of a mouse model of hyper-IgM X-linked immunodeficiency. *Nat Med* **10**, 835-841 (2004).

4 He, X. X. *et al.* MicroRNA-375 targets AEG-1 in hepatocellular carcinoma and suppresses liver cancer cell growth in vitro and in vivo. *Oncogene* **31**, 3357-3369 (2012).

5 Henglein, B., Chenivesse, X., Wang, J., Eick, D. & Brechot, C. Structure and cell cycle-regulated transcription of the human cyclin A gene. *Proc Natl Acad Sci U S A* **91**, 5490-5494 (1994).

6 Di Agostino, S. *et al.* Gain of function of mutant p53: the mutant p53/NF-Y protein complex reveals an aberrant transcriptional mechanism of cell cycle regulation. *Cancer cell* **10**, 191-202 (2006).

7 Tetsu, O. & McCormick, F. Beta-catenin regulates expression of cyclin D1 in colon carcinoma cells. *Nature* **398**, 422-426 (1999).

8 Geng, Y. *et al.* Regulation of cyclin E transcription by E2Fs and retinoblastoma protein. *Oncogene* **12**, 1173-1180 (1996).

9 el-Deiry, W. S. *et al.* WAF1, a potential mediator of p53 tumor suppression. *Cell* **75**, 817-825 (1993).

10 Duan, H., Heckman, C. A. & Boxer, L. M. The immunoglobulin heavy-chain gene 3' enhancers deregulate bcl-2 promoter usage in t(14;18) lymphoma cells. *Oncogene* **26**, 2635-2641 (2007).

11 Yu, J., Zhang, L., Hwang, P. M., Kinzler, K. W. & Vogelstein, B. PUMA induces the rapid apoptosis of colorectal cancer cells. *Molecular cell* **7**, 673-682 (2001).
